# Medical Clinic for Session of 1860

**Published:** 1860-11

**Authors:** J. G. Westmoreland

**Affiliations:** Atlanta, Ga.


### *Medical Clinic for the Session of* 1860. By J. G. WESTMORELAND, M. D., of Atlanta, Ga.

[Continued from page 85.]

JUNE 5.—Negro woman æt. 30 years, the mother of several children, the youngest of which is now one year old.—This is a case of mania, or perhaps, may be more properly termed dementia. The reasoning faculties, judgment and emotions of the mind are almost entirely destroyed. Mania is characterized by pervertion of the judgment, illusion, and may exist with the ordinary perfection of the reasoning faculties. The perceptions are deranged so that the subject of mania labors under illusions and hallucinations, and supposing these impressions real, the subject reasons and acts rationally upon them. In the case before us there is an evident want of sensibility and thought—a want of mind. This disease is of two months' standing; commencing gradually, progressed for a month before the degree of mental inactivity now found, was produced. In contradistinction to congenital dementia or idiotcy, this variety of mental derangement is called "dementia accidentalis."

We have a very imperfect history of this case, but there are some reasons for supposing this woman has been the subject of such attacks previously, and that they came on during pregnancy. While there is no positive evidence of pregnancy now, well grounded suspicions may be entertained of her having conceived. The absence of catamenial discharge for two months, so far as it goes, favors this supposition. I hope soon to obtain a more reliable history of her previous attacks, and to get more satisfactory evidences of the true condition of the uterus.

The treatment adopted, and which has been applied for a week, consists of 1 gr. ex't. hyoscyamus three times a day, and the shower-bath every morning. No favorable impression has yet been made.

JUNE 7.—*Negro woman thirty-five years old—Dysmenorrhœa.*

That state of the uterus in which we find painful menstruation, is unfavorable to conception, though the periods may appear regularly. The various forms of disturbance of the system, consequent upon reflex nervous action, are also to be found where dysmenorrhœa is a prominent symptom.— This state of things exists often when no organic lesion of the womb can be detected, and doubtless is the result of functional derangement, produced by a want of the proper nervous energies of the uterus and its appendages.

Those remedies, called emmenagogues, which give strength to the nervous energies, are proper under such circumstances for permanent relief. There are strammonium, guaiac, &c.

Nervous stimulants, such as musk, amber, assafœtida, &c., counteract reflex nervous influences—hysteria—for the time, but have no permanent impression or tendency to restore the normal condition of the organ primarily disturbed. This case was before you on a former occasion, when laboring under a partial paralysis of one of the upper extremities, as one of the results of reflex nervous influences from disturbance of the uterus. That unpleasant condition passed away under action of the prescription then made—counter irritants to the spine, nervous stimulants, &c.

June 9.—*Chronic Laryngitis—Clergyman's Sore Throat. —White man 50 years old.*

We had occasion to call attention to pectoral diseases, some time ago, when there was presented for examination, a case of phthisis pulmonalis. We now exhibit a case of probable tubercular disease, in the upper portion of the respiratory tube particularly, and extending, perhaps, to the lungs themselves. In this case, differing from the one referred to, we have no very distinct physical evidences of pulmonic lesion. Disturbance of the voice, amounting to almost complete aphony, is the most prominent symptom.—The emaciation and other evidences of an enfeebled condition of the system, shows disturbance of the nutritive function, and connects constitutional depression with the local disease of the air passages, either as the cause or result.

The prognosis in this case, must depend upon the opinion entertained of the cause of local disturbance, whether from tuberculous deposite or other source of irritation. If tuberculous matter be deposited in the form of miliary tubercles, the only hope of success is in invigorating the whole physical organism, so that a point of energy is attained above that favorable to the deposite of this degenerate substance.

The only rational course of treatment is easily arrived at, and readily applied. Constitutional support and invigoration, by nourishing diet, wine, opium, &c., is of the first importance. Local applications of solution nitrate of silver, fumes of iodine, &c., to the larynx and other portions of the air passages, are useful generally, but curative only when the local disease does not depend on tuberculous deposite, or when the deposite has ben arrested. The application to the larynx may be best made with the probang, but where the disease exists in the bronchia also, the inhalation of the fumes of iodine is preferable.

